# Effect of Kunxian capsule, a novel *Tripterygium wilfordii* preparation, on reducing proteinuria and slowing renal function decline in diabetic kidney disease: a 48–week randomized controlled trial

**DOI:** 10.1080/0886022X.2026.2755136

**Published:** 2026-11-04

**Authors:** Shengnan Chen, Yajuan Gao, Zishen Liang, Yixue Zhang, Cui Wang, Yali Tang, Jun Zhao, Ani Wang, Yan Shen

**Affiliations:** ^a^ Department of Nephrology, The First Affiliated Hospital of Xi’an Jiaotong University, Xi’an, China; ^b^ Department of Computing, The Hong Kong Polytechnic University, Hong Kong, China

**Keywords:** Diabetic kidney disease, Kunxian capsule, proteinuria, renal function, renal composite endpoints, irbesartan

## Abstract

**Background:**

Diabetic kidney disease (DKD), a leading cause of chronic kidney disease, remains a clinical challenge despite renin–angiotensin system blockade. This study evaluated the efficacy and safety of adding Kunxian capsule to standard therapy with irbesartan in patients with DKD.

**Methods:**

Eighty patients with DKD were randomly assigned to either the experimental group (standard therapy with irbesartan plus Kunxian capsule) or the control group (standard therapy with irbesartan) for 48 weeks. Primary outcomes included the proteinuria remission rate, degree of proteinuria reduction, and median time to remission. Secondary outcomes included the slope of eGFR decline and the incidence of renal composite hard endpoint. Safety was assessed by monitoring adverse events. This trial was registered in ClinicalTrials.gov on March 2, 2021 (No. NCT04981613).

**Results:**

In the intention–to–treat analysis, the experimental group had a higher proteinuria remission rate (57.50% *vs.* 25.00%), a shorter median time to remission (5 months *vs.* not reached), and a greater reduction in 24–hour urinary protein than the control group. A lower incidence of renal composite hard endpoint was observed in the experimental group (10.00% *vs.* 32.50%), accompanied by a slower eGFR decline. No significant differences in the incidence of adverse events were observed between the two groups. Inflammatory markers, including C–reactive protein and erythrocyte sedimentation rate, were significantly reduced in the experimental group.

**Conclusions:**

Adding Kunxian capsule to standard therapy with irbesartan improved the proteinuria remission rate and attenuated renal function decline in patients with DKD, supporting its potential as a promising adjunctive therapy.

## LAY SUMMARY

Diabetic kidney disease (DKD) is a serious complication of diabetes that can cause proteinuria and progressive loss of kidney function. Although current medications help protect the kidneys, many patients still experience persistent proteinuria and worsening kidney function. In this 48–week clinical trial involving 80 patients, researchers evaluated whether adding Kunxian capsule – a traditional Chinese medicine formulation – to standard therapy with irbesartan could improve outcomes. The results showed that patients who took Kunxian capsule alongside standard therapy with irbesartan achieved a higher rate of proteinuria remission and a greater reduction in proteinuria, experienced a slower decline in kidney function, and had fewer composite renal events compared with those receiving irbesartan alone. These findings suggest that Kunxian capsule may be a promising adjunctive treatment option to help reduce proteinuria and protect kidney function in patients with DKD.

## KEY MESSAGES

1. What is known: Despite advances in kidney–protective therapies, persistent proteinuria and progressive loss of kidney function remain major clinical challenges in patients with DKD.
2. This study adds: In this 48–week randomized controlled trial, patients receiving standard therapy with irbesartan plus Kunxian capsule had a higher proteinuria remission rate, greater proteinuria reduction, a slower decline in eGFR, and fewer composite renal events than those receiving standard therapy with irbesartan alone.
3. Potential impact: These findings provide prospective clinical evidence supporting Kunxian capsule as a promising adjunctive treatment option for DKD.

## GRAPHICAL ABSTRACT

**Figure:**
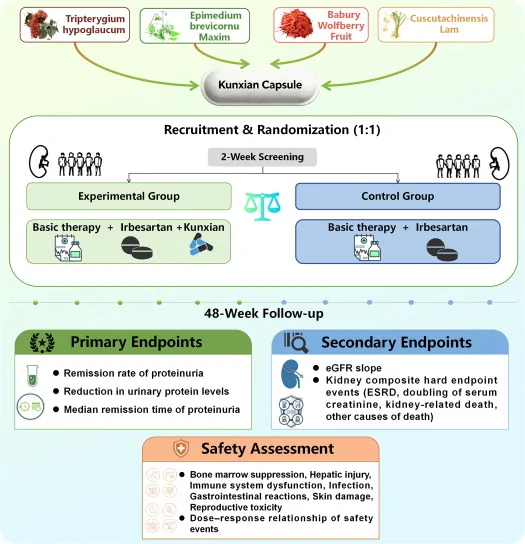

## 1. Introduction

The global prevalence of diabetes continues to rise, and its major microvascular complication – diabetic kidney disease (DKD) – has become the leading cause of chronic kidney disease (CKD) and end–stage renal disease (ESRD), imposing a substantial healthcare and socioeconomic burden worldwide [1]. The hallmark pathological changes in DKD include glomerular basement membrane thickening and mesangial matrix expansion [2]. These alterations contribute to proteinuria primarily by increasing glomerular capillary permeability and disrupting the negative charge barrier [3]. Consequently, progressive proteinuria is not only the most characteristic clinical manifestation of DKD but also a reliable predictor of adverse renal outcomes. Large–scale meta–analyses have confirmed that early changes in albuminuria can serve as a surrogate endpoint for renal function decline, underscoring the clinical importance of effective proteinuria reduction in delaying DKD progression [4].

Currently, renin–angiotensin–aldosterone system (RAAS) inhibitors, including angiotensin-converting enzyme inhibitors and angiotensin receptor blockers, are recommended as a cornerstone of treatment for DKD [5]. They exert renoprotective effects by lowering systemic blood pressure, ameliorating glomerular hyperfiltration, and suppressing inflammation and fibrosis driven by RAAS activation, thereby reducing proteinuria and slowing renal function deterioration [6,7]. In the Irbesartan Diabetic Nephropathy Trial, irbesartan treatment resulted in a 33% reduction in proteinuria, a 33% decrease in the risk of serum creatinine doubling, and a 23% decline in the incidence of ESRD [8]. Beyond RAAS blockade, sodium–glucose cotransporter 2 (SGLT2) inhibitors have become an important component of kidney-protective therapy for patients with type 2 diabetes [9]. Their renal benefits extend beyond glucose lowering and include reductions in albuminuria, intraglomerular pressure, and estimated glomerular filtration rate (eGFR) decline, thereby reducing the risk of CKD progression [10,11]. Finerenone, a nonsteroidal mineralocorticoid receptor antagonist, has also been shown to reduce the risk of CKD progression and cardiovascular events in patients with type 2 diabetes and albuminuric CKD receiving maximally tolerated RAAS blockade [12,13]. Despite these advances, residual proteinuria and progressive renal dysfunction remain important clinical challenges, particularly in patients receiving standard kidney-protective therapy. Therefore, additional adjunctive strategies for DKD warrant further investigation.

In this context, traditional Chinese medicine (TCM) compound formulations are gaining increasing attention in kidney disease management due to their multi-target and multi-pathway synergistic effects. Kunxian capsule, a representative achievement of the National ‘Ninth Five-Year Plan’ Key Scientific and Technological Research Project in TCM, is a third-generation upgraded compound preparation based on *Tripterygium hypoglaucum* as the principal component, combined with *Epimedium brevicornu, Lycium barbarum,* and *Cuscuta chinensis* [14]. Modern pharmacological studies have demonstrated that Kunxian capsule possesses potent immunomodulatory, anti-inflammatory, and antioxidant properties [14,15]. Clinically, it has shown promising efficacy in modulating immune-inflammatory status, reducing proteinuria, and delaying renal function decline across various glomerular diseases, including lupus nephritis, Henoch-Schönlein purpura nephritis, membranous nephropathy, and IgA nephropathy [16–19].

Importantly, the pharmacological properties of Kunxian capsule align with the core pathophysiological hallmarks of DKD identified in our previous research, including inflammation, immune cell infiltration, and oxidative stress [20–22]. This provides a theoretical basis and promising rationale for Kunxian capsule to further reduce proteinuria and delay disease progression in DKD through non-hemodynamic mechanisms. Consistent with this hypothesis, animal studies conducted by Jin et al. demonstrated that Kunxian capsule exerts renoprotective effects by inhibiting the β-catenin signaling pathway [23]. Given that existing evidence is largely restricted to small-sample observational or mechanistic investigations, there is an urgent need for well-designed and prospective clinical trials to rigorously evaluate the long-term efficacy and safety profile of Kunxian capsule in DKD.

Therefore, we conducted a randomized, open-label, parallel-controlled prospective study to comprehensively evaluate the efficacy of Kunxian capsule as an adjunct to irbesartan-based therapy in promoting proteinuria remission, preserving renal function, and improving long-term renal outcomes in patients with DKD. In addition, adverse events were systematically monitored throughout the treatment period to further characterize the safety profile of Kunxian capsule in clinical use. The findings of this study are expected to provide clinical evidence for the potential use of Kunxian capsule as an adjunctive therapy for DKD and inform the development of more effective therapeutic strategies for improving renal outcomes and reducing disease burden.

## 2. Methods

### 2.1. Study design

This study was a randomized, open-label, parallel-controlled, prospective trial. The open-label design was selected because the primary efficacy outcomes were largely objective and it facilitated routine safety monitoring and management of adverse events during the follow-up period. Participants were patients with DKD who met the inclusion and exclusion criteria at the Department of Nephrology in the First Affiliated Hospital of Xi’an Jiaotong University. The diagnosis of DKD was established by nephrologists based on a comprehensive clinical assessment in accordance with the American Diabetes Association criteria. Specifically, DKD was clinically defined as an eGFR < 60 mL/min/1.73 m^2^ or a urine albumin-to-creatinine ratio (UACR) > 30 mg/g persisting for more than 3 months [24]. Patients meeting these clinical criteria were screened, and those with pathologically confirmed DKD based on available kidney biopsy findings were included. After a 2-week screening period, during which irbesartan was initiated at a dose of 0.15 g once daily and gradually titrated to the target dose of 0.3 g once daily in the morning, patients who tolerated the target dose without experiencing drug-related adverse events were enrolled. Eligible participants were randomly assigned in a 1:1 ratio to either the experimental group (Kunxian capsule plus irbesartan) or the control group (irbesartan alone) using a computer-generated random number table. The personnel responsible for participant recruitment and intervention assignment were blinded to the random allocation sequence. The planned treatment and follow-up period was 48 weeks, with scheduled visits every 4 weeks. All participants in both groups received standard-of-care treatment for DKD in accordance with the 2020 Kidney Disease: Improving Global Outcomes clinical practice guidelines [7]. Kunxian capsule was administered at a dose of 0.6 g, three times daily, orally with meals. The first visit was conducted at the end of the screening period (Week −2), followed by scheduled visits every 4 weeks. At each scheduled visit, blood and urine samples were collected, and primary and secondary endpoints, as well as safety events, were systematically recorded.

The prespecified co-primary outcomes were proteinuria remission rate, degree of proteinuria reduction, and time to proteinuria remission. Treatment response was categorized as follows: complete remission (CR), defined as 24-h urinary protein (24h-UP) ≤ 0.3 g; partial remission (PR), defined as a 24h-UP > 0.3 g but < 3.5 g with a reduction of at least 50% from baseline; and no remission (NR), defined as a reduction in 24h-UP of less than 50% from baseline. Proteinuria remission was defined as the achievement of either CR or PR at a scheduled study visit, based on a single measurement. The remission time was defined as the first scheduled visit at which the criteria for CR or PR were met. The degree of proteinuria reduction was assessed based on changes in 24h-UP, calculated as the level at each follow-up visit minus the baseline level. Secondary outcomes included the slope of eGFR decline and a renal composite hard endpoint, defined as the occurrence of ESRD requiring dialysis or kidney transplantation, doubling of serum creatinine, renal-related death, or other causes of death. Safety assessments focused on comparing the incidence and timing of adverse events between the two groups, including bone marrow suppression, hepatotoxicity, immunotoxicity, infections, gastrointestinal reactions, dermatologic injury, and reproductive toxicity.

This trial was registered in ClinicalTrials.gov on March 2, 2021 (Registration No. NCT04981613) and was also recorded in the Medical Research Registration and Filing Information System on July 16, 2021 (Registration No. MR-61-21-014620). The study was conducted from July 28, 2021 to August 28, 2024. The protocol adhered to the Declaration of Helsinki and was approved by the Ethics Committee of the First Affiliated Hospital of Xi’an Jiaotong University (Approval No. XJTU1AF2021LSK-220). Written informed consent was obtained from all participants. The study design and participant flow are presented in Figure 1.

**Figure 1.**
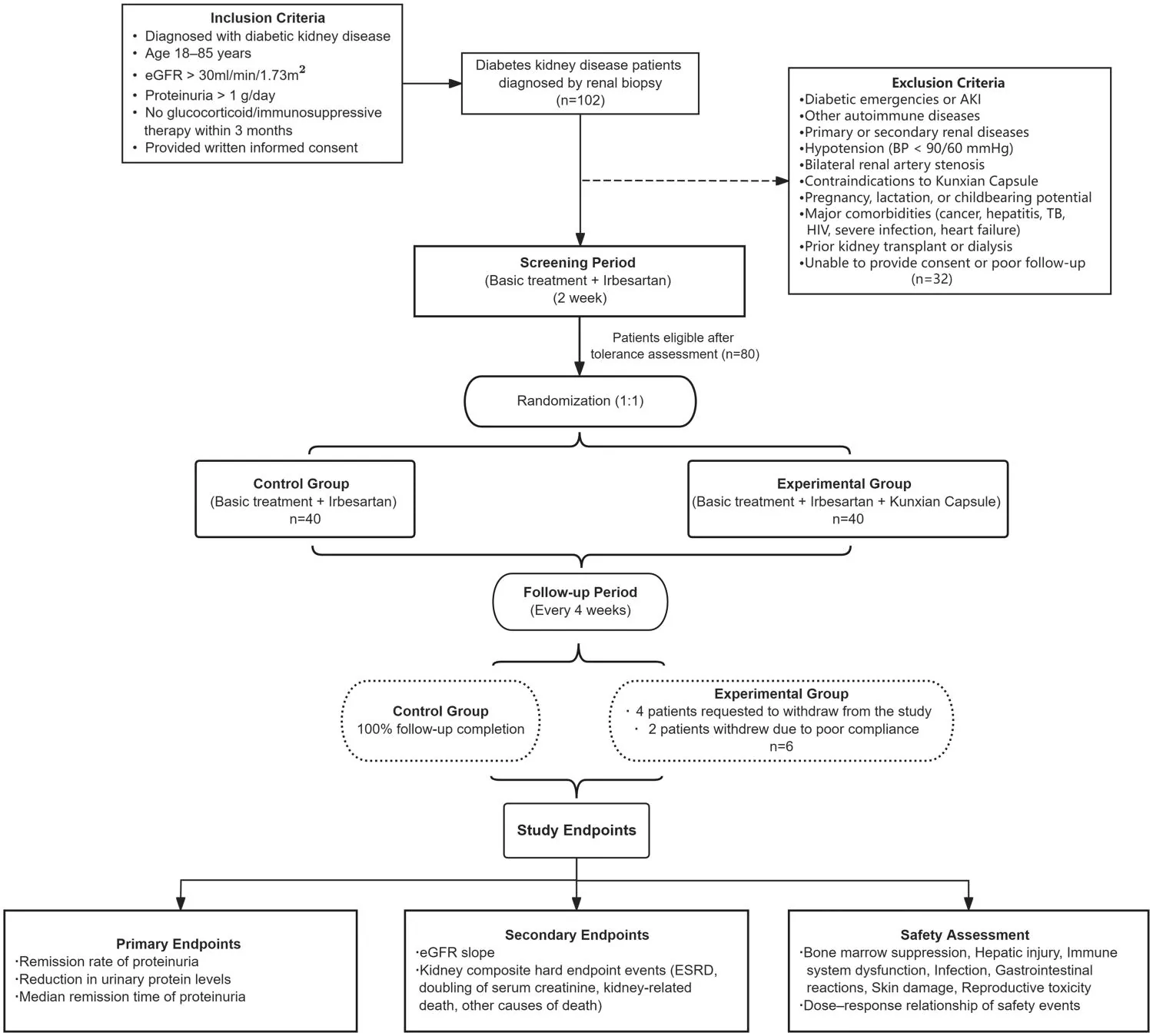
Flow diagram of study participants.

### 2.2. Statistical analysis

Based on previous studies, a between-group difference of 2.5 g in 24h-UP reduction was assumed as the expected effect size [25]. A superiority trial with a 1:1 parallel design was adopted. The sample size was calculated using PASS software. Assuming a one-sided α of 0.025 and a power (1 − β) of 80%, a minimum of 18 patients per group was required to detect the predefined difference between groups. No interim analyses or formal stopping rules were planned or conducted during the trial. Analyses of endpoints were conducted using the intention-to-treat (ITT) and per-protocol (PP) approaches. The prespecified primary outcomes were analyzed as co-primary endpoints; therefore, no hierarchical testing procedure or multiplicity adjustment was prespecified. For baseline characteristics, continuous variables were assessed for normality using the Shapiro-Wilk test and for homogeneity of variance using Levene’s test. Data conforming to a normal distribution with homogeneous variances were expressed as mean ± standard deviation ($\bar{x}$± s) and compared between groups using the independent-samples t-test. Non-normally distributed data were presented as median and interquartile range (M, IQR) and compared using the Mann-Whitney U test. Categorical variables were summarized as counts and percentages and compared using the chi-square test or Fisher’s exact test.

Kaplan-Meier survival analysis was employed to compare the cumulative incidence of proteinuria remission and renal composite hard endpoint between the two groups, with between-group differences assessed using the log-rank test. Cox proportional hazards regression models were used to estimate hazard ratio (HR) and 95% confidence interval (CI) for the renal composite hard endpoint. To evaluate the cumulative risk of safety events, scatter plots were used to visualize the distribution of adverse event onset times in relation to cumulative treatment duration.

A linear mixed-effects model (LMM) was used to assess longitudinal changes in eGFR and to estimate the eGFR slope. Fixed effects included time (week), treatment group, the time-by-treatment group interaction, age, sex, and baseline eGFR. The model included subject-specific random intercepts and random slopes for time, with an unstructured variance-covariance matrix specified for the random effects. The residuals were assumed to be independently and identically distributed. Model parameters were estimated using restricted maximum likelihood, and degrees of freedom were calculated using the Satterthwaite approximation. The time-by-treatment group interaction was used to assess the overall difference in the rate of eGFR change between the two groups. Adjusted mean eGFR slopes and corresponding 95% CIs were estimated using the estimated marginal means framework, and between-group differences in slopes were assessed using a t-test. Individual eGFR slopes were derived from the empirical Bayes estimates of the subject-specific random effects. Missing eGFR values were not imputed. The distribution of individual eGFR slopes was visualized using a ridge plot.

All statistical analyses were performed using R statistical software. All tests were two-sided, and a *P* value < 0.05 was considered statistically significant.

## 3. Results

### 3.1. Baseline characteristics and study flow

A total of 80 patients with DKD were enrolled based on the predefined inclusion and exclusion criteria and randomly assigned to either the experimental group (*n* = 40) or the control group (*n* = 40). During the follow-up period, no premature withdrawals occurred in the control group, yielding a follow-up completion rate of 100%. In contrast, six patients in the experimental group discontinued the study: four patients discontinued follow-up because of difficulties traveling to the study site, while two patients discontinued because of poor treatment adherence and incomplete follow-up data. Ultimately, 74 patients completed the study. Baseline characteristics were compared between patients who completed the follow-up and those who discontinued the study (Supplementary Table 1), and no significant differences were observed. Baseline demographic, clinical, and laboratory characteristics were well balanced between the experimental and control groups (Table 1).

**Table 1.** Comparison of baseline characteristics between the experimental and control groups.

Characteristics	Control group (*n* = 40)	Experimental group (*n* = 40)	*P* value
Age (year, $\bar{x}$ ± s)	52.075 ± 12.200	54.175 ± 11.190	0.425
Sex (n, male/female)	25/15	27/13	0.639
Body mass index (kg/m^2^, $\bar{x}$ ± s)	24.697 ± 3.085	25.020 ± 2.984	0.643
CKD Stage (n, Stage 1/2/3)	10/13/17	13/10/17	0.676
Systolic blood pressure (mmHg, $\bar{x}$ ± s)	129.150 ± 14.913	126.600 ± 9.668	0.367
Fasting plasma glucose (mmol/L, (M, IQR))	6.950, 2.865	6.380, 1.990	0.874
2-hour plasma glucose (mmol/L, (M, IQR))	10.000, 3.960	10.000, 1.613	0.761
Glycated hemoglobin (%, (M, IQR))	7.450, 2.725	7.150, 1.750	0.754
24-hour urinary protein (g/d, (M, IQR))	4.010, 3.556	3.580, 2.935	0.131
UACR (mg/g, (M, IQR))	3195.760, 3631.560	2587.230, 1711.550	0.056
Albumin (g/L, (M, IQR))	34.600, 7.430	34.600, 8.250	0.596
Blood urea nitrogen (mmol/L, (M, IQR))	7.380, 3.425	8.090, 4.625	0.281
Serum creatinine (μmol/L, (M, IQR))	111.000, 66.500	108.000, 61.250	0.447
Cystatin C (mg/L, (M, IQR))	1.738, 0.769	1.686, 0.883	0.920
Serum uric acid (μmol/L, (M, IQR))	362.000, 137.000	384.000, 85.250	0.954
eGFR (mL/min/1.73 m², (M, IQR))	55.710, 42.120	60.445, 51.400	0.693
Triglycerides (mmol/L, (M, IQR))	1.650, 0.895	1.690, 1.840	0.821
Total cholesterol (mmol/L, (M, IQR))	5.300, 2.115	5.175, 1.675	0.543
Fibrinogen (g/L, (M, IQR))	4.600, 1.870	4.775, 1.908	0.630
Erythrocyte sedimentation rate (mm/h, (M, IQR))	45.500, 47.000	34.000, 29.250	0.337
C-reactive protein (mg/L, (M, IQR))	1.805, 2.053	1.280, 2.583	0.384
Helper T-cell count (cells/μL, (M, IQR))	637.510, 360.285	623.940, 336.665	0.256
Immunoglobulin G (g/L, (M, IQR))	8.205, 4.330	9.860, 4.548	0.104
Metformin Administration (n, (Yes, No))	18, 22	23, 17	0.263
SGLT2 Inhibitor Administration (n, (Yes, No))	36, 4	37, 3	1.000
GLP-1R Agonist Administration (n, (Yes, No))	2, 38	5, 35	0.432
DPP-4 Inhibitor Administration (n, (Yes, No))	8, 32	5, 35	0.363
Insulin Administration (n, (Yes, No))	24, 16	20, 20	0.369
Atorvastatin Administration (n, (Yes, No))	23, 17	24, 16	0.820
MRA Administration (n, (Yes, No))	0, 40	0, 40	–

Annotations: CKD, chronic kidney disease; UACR, urinary albumin-to-creatinine ratio; eGFR, estimated glomerular filtration rate; SGLT2, sodium-glucose cotransporter 2; GLP-1R, glucagon-like peptide-1 receptor; DPP-4, dipeptidyl peptidase-4; MRA, mineralocorticoid receptor antagonist.

### 3.2. Primary outcome analysis

The analysis of the primary outcome demonstrated that, in both the ITT and PP populations, the proteinuria remission rate was significantly higher in the experimental group than in the control group (Table 2). Kaplan-Meier analysis showed that the median time to proteinuria remission was 5.0 months in the experimental group and was not reached in the control group during the follow-up period in the ITT population (*p* < 0.001, Figure 2A). Consistent results were observed in the PP population, with a median time to proteinuria remission of 4.5 months in the experimental group, whereas the median was not reached in the control group (*p* < 0.0001, Figure 2B). In addition, the median change in 24h-UP indicated better proteinuria control in the experimental group than in the control group, with significant between-group differences in both the ITT and PP populations (Table 2).

**Figure 2.**
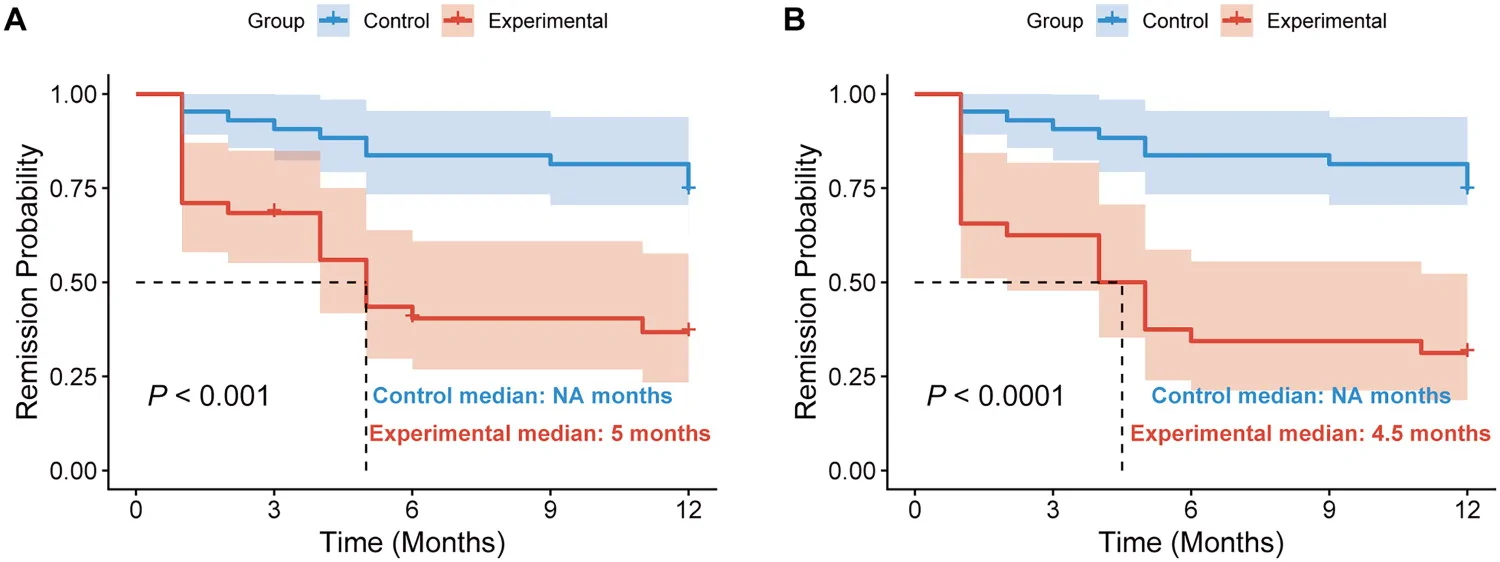
Kaplan-Meier analysis of time to proteinuria remission in the (A) ITT and (B) PP populations.

**Table 2.** Intention-to-treat and per-protocol analyses of primary and secondary outcomes.

	ITT analysis (*n* = 80)			PP analysis (*n* = 74)		
Outcome	Control group (*n* = 40)	Experimental group (*n* = 40)	*P* value	Control group (*n* = 40)	Experimental group (*n* = 34)	*P* value
**Primary outcomes**						
Proteinuria remission rate (n, %)	10, 25.00	23, 57.50	<0.0001	10, 25.00	23, 67.65	<0.001
Median time to proteinuria remission (months)	Not reached	5.000	<0.001	Not reached	4.500	<0.0001
Change in 24h-UP (g/d, M, IQR)	0.460, 0.810	0.000, 2.000	0.048	0.460, 0.810	−1.000, 3.000	0.019
**Secondary outcomes**						
Renal composite endpoint (n, %)	13, 32.50	4, 10.00	0.039	13, 32.50	4, 11.76	0.042
eGFR slope (mL/min/1.73 m² per week, adjusted mean [95% CI])	−0.547 [-0.720 to −0.374]	−0.266 [-0.443 to −0.089]	0.027	−0.547 [-0.720 to −0.374]	−0.268 [-0.448 to −0.088]	0.030

### 3.3. Secondary outcome analysis

#### 3.3.1. Comparison of renal composite hard endpoint

During the follow-up period, the cumulative incidence of the renal composite hard endpoint was lower in the experimental group than in the control group in both the ITT and PP populations (Figure 3A and 3B). Specifically, in the ITT population, the cumulative incidence was 10.00% in the experimental group compared with 32.50% in the control group. Cox regression analysis showed that Kunxian capsule treatment was associated with a lower risk of the renal composite hard endpoint (HR, 0.323; 95% CI, 0.105–0.991; *p* = 0.048). The individual components of the composite endpoint are reported in Supplementary Table 2.

**Figure 3.**
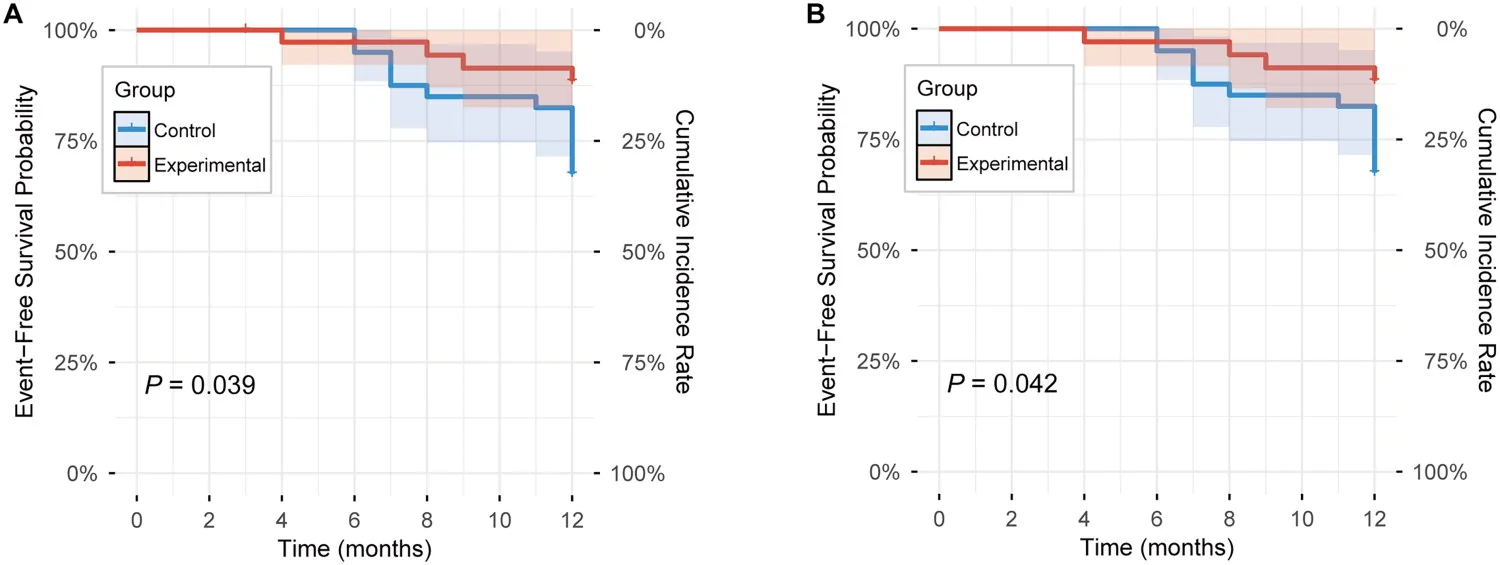
Cumulative incidence of the renal composite hard endpoint in the (A) ITT and (B) PP populations.

#### 3.3.2. eGFR slope and individual trajectories

Individual eGFR trajectories illustrated the longitudinal changes in renal function over the 48-week follow-up, with a less pronounced decline in the experimental group than in the control group (Figure 4A). Consistent with these trajectories, the adjusted mean eGFR slope estimated using an LMM (adjusted for sex, age, and baseline eGFR) was less negative in the experimental group than in the control group (Figure 4B), indicating a slower rate of eGFR decline during follow-up.

**Figure 4.**
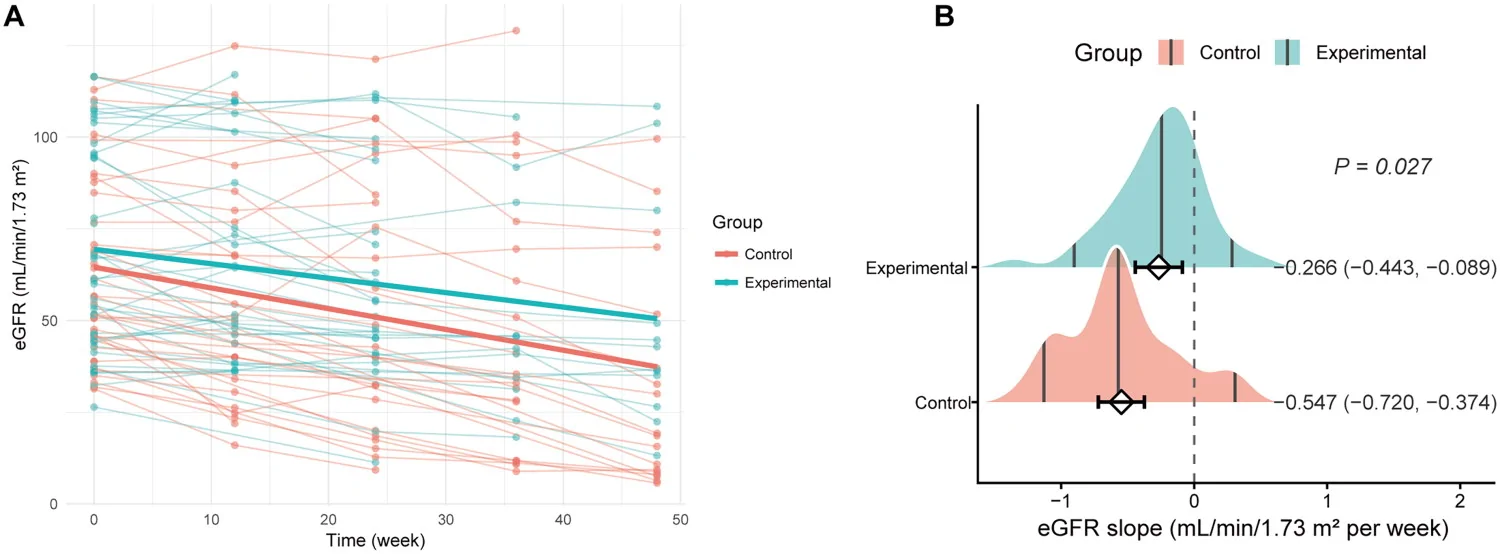
Longitudinal (A) eGFR trajectories and (B) adjusted eGFR slopes in the experimental and control groups.

### 3.4. Safety outcomes and adverse events

A scatter plot depicting the timing of adverse events in relation to treatment duration showed similar distributions of event occurrence and onset time between the two groups. There was no significant difference in the cumulative incidence of adverse events between the experimental and control groups (27.50% *vs.* 30.00%, *p* > 0.05) (Figure 5A). In the experimental group, reported adverse events included urinary tract infection (7.50%), leukopenia (2.50%), herpes zoster infection (2.50%), approximately one-fold elevation of liver transaminases (7.50%), hyperkalemia (2.50%), acute heart failure (2.50%), and menstrual disorders (2.50%). Adverse events observed in the control group comprised urinary tract infection (7.50%), thrombocytopenia (2.50%), pulmonary infection (2.50%), approximately one-fold elevation of liver transaminases (2.50%), hyperkalemia (2.50%), acute heart failure (5.00%), fatal severe skin and soft tissue infection (2.50%), and fatal intracerebral hemorrhage (5.00%). Assessment of immune function revealed no statistically significant differences in helper T-cell counts or serum IgG levels between the experimental and control groups (Figure 5B–C).

**Figure 5.**
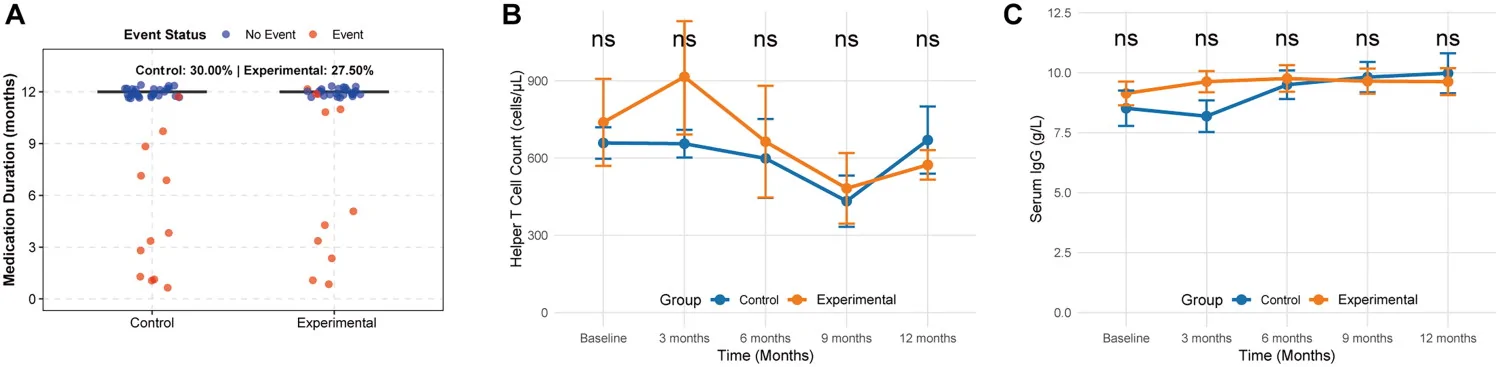
Safety assessment in the experimental and control groups: (A) cumulative incidence of adverse events, (B) helper T-cell counts, and (C) serum IgG levels.

### 3.5. Effects of kunxian capsule on inflammatory markers

To evaluate the effects of Kunxian capsule on systemic inflammatory status, erythrocyte sedimentation rate (ESR) and C-reactive protein (CRP) levels were compared between the two groups. The results showed that ESR and CRP levels were significantly lower in the experimental group than in the control group during follow-up (Supplementary Figure 1).

## 4. Discussion

DKD has become a major global health burden. Although the introduction of novel glucose-lowering agents has substantially improved patient outcomes, the prevalence of the disease and the risk of disease progression remain high [26]. Therefore, exploring additional therapeutic strategies is crucial for improving renal prognosis in DKD. In this randomized controlled trial, we found that adding Kunxian capsule to standard therapy with irbesartan was associated with a greater reduction in 24-h UP, a higher proteinuria remission rate, and a slower rate of eGFR decline, with a lower incidence of renal composite hard endpoints observed during follow-up. These findings provide clinical evidence supporting Kunxian capsule as a potential adjunctive therapy for DKD.

Proteinuria is an important therapeutic target in DKD, as persistent proteinuria is closely associated with subsequent decline in renal function and disease progression. The benefits observed in the present study should also be interpreted in the context of concomitant kidney-protective therapies. Given the established renoprotective effects of SGLT2 inhibitors, their use was considered a potential factor influencing the observed outcomes. At baseline, SGLT2 inhibitor use was similar between the experimental and control groups. Although SGLT2 inhibitor use varied during follow-up, no statistically significant between-group differences were observed at any scheduled follow-up visit (Supplementary Table 3). Therefore, the additional benefits observed in the experimental group may be attributable to Kunxian capsule treatment.

Previous studies have suggested that the therapeutic effects of Kunxian capsule may involve the pharmacological activities of *Tripterygium hypoglaucum* and its bioactive constituents, together with the complementary effects of its other herbal components [14,27,28]. Animal studies in DKD models have demonstrated that triptolide attenuates proteinuria by suppressing macrophage infiltration, alleviating podocyte injury, and inhibiting the expression of TGF-β1 and MCP-1 [27, 28]. Furthermore, another animal study in DKD has indicated that Kunxian capsule may ameliorate renal structural and functional abnormalities through modulation of the Wnt/β-catenin signaling pathway [23]. Insights from other disease models reinforce these findings. In a lupus nephritis model, Kunxian capsule was reported to inhibit JAK1/STAT1 signaling and reduce T-cell infiltration, further supporting its potential role in regulating renal inflammation and immune responses [16]. In rheumatoid arthritis, mechanistic studies have also suggested that Kunxian capsule may modulate the PI3K/AKT/mTOR pathway and inhibit the NF-κB/IKK inflammatory network, thereby reducing the production of pro-inflammatory cytokines [14]. In the present study, patients receiving Kunxian capsule treatment achieved earlier proteinuria remission and exhibited lower levels of inflammatory markers (CRP and ESR) starting as early as 3 months. These clinical findings are highly consistent with previously reported anti-inflammatory and podocyte-protective effects of Kunxian capsule.

To reduce the toxicity associated with traditional *Tripterygium wilfordii* preparations, Kunxian capsule was formulated with *Epimedium*, *Cuscuta chinensis*, and *Lycium barbarum*. These herbs contain bioactive constituents with reported anti-inflammatory, immunomodulatory, antioxidant, and organ-protective activities, and their complementary actions may contribute to the overall pharmacological profile of the formulation [29–34]. Nevertheless, the safety profile of Kunxian capsule remains an important consideration in clinical application. Reported adverse reactions associated with Kunxian capsule include bone marrow suppression, gastrointestinal disturbances, reproductive toxicity, hepatic dysfunction, renal injury, and skin lesions [14,15]. In the present study, the cumulative incidence of adverse events did not differ significantly between the two groups, and no statistically significant associations were detected between the incidence or timing of adverse events and the cumulative dosage or duration of Kunxian capsule use. The most common adverse reactions in the experimental group were abnormal liver function and urinary tract infections. The occurrence of urinary tract infections may be related to the concomitant use of SGLT2 inhibitors in some patients, whereas the observed hepatic dysfunction highlights the necessity of regular liver function monitoring in clinical practice. Moreover, the anti-fertility effects of *Tripterygium wilfordii* preparations have been confirmed in animal studies [35]. Previous research has indicated that approximately 11.70% of patients may have experienced reproductive-related adverse events [36]. In this study, only one case of menstrual disorder was reported in the Kunxian capsule group. This may be attributed to the lower proportion of enrolled female patients, most of whom were postmenopausal, and a potential antagonistic effect of *Lycium barbarum* against the reproductive toxicity of *Tripterygium hypoglaucum*. Overall, Kunxian capsule exhibits an acceptable safety profile in patients with DKD. Nevertheless, clinicians should exercise caution, particularly in patients with preexisting hepatic impairment or hypoalbuminemia, as well as in women of childbearing potential.

A major barrier to the widespread clinical translation of many TCM formulations is the lack of high-quality clinical evidence. A key strength of the present study lies in its prospective design and the extended follow-up duration of up to 1 year for each participant. This design allowed the evaluation of treatment responses and safety outcomes over a relatively prolonged period, thereby adding prospective clinical evidence to the existing literature on Kunxian capsule in DKD. Nevertheless, this study remains exploratory in nature and has several limitations. First, the open-label design may have introduced bias potentially favoring the experimental group and could have overestimated the treatment effect. However, the primary outcomes were largely based on objective measures, which helped reduce subjective outcome assessment bias. Second, this was a single-center study with a relatively small sample size. The limited sample size and number of renal composite hard endpoints, both overall and within individual subgroups, constrained the statistical power for subgroup analyses stratified by CKD stage or albuminuria level. Consequently, these findings should be interpreted with caution and warrant further confirmation in future large-scale, multicenter randomized clinical trials.

## 5. Conclusions

In summary, this study indicates that adding Kunxian capsule to irbesartan increases the proteinuria remission rate and reduces proteinuria levels in patients with DKD. Exploratory analyses also suggested a slower rate of eGFR decline and a lower incidence of renal composite hard endpoints in the Kunxian capsule group. These findings help bridge the current gap in clinical evidence for TCM in DKD and provide evidence-based support for Kunxian capsule as a potential adjunctive therapy for DKD. Moreover, this study offers a novel therapeutic perspective for improving renal outcomes in patients with DKD.

## Supplementary Material

Supplementary material.docx

## Data Availability

The data analyzed during the current study are not publicly available due to patient privacy concerns, but are available from the corresponding author on reasonable request.
