# Supplementary material for: Effect of Kunxian capsule, a novel Tripterygium wilfordii preparation, on reducing proteinuria and slowing renal function decline in diabetic kidney disease: a 48–week randomized controlled trial
Source: Ren Fail. 2026 Nov 4;48(1):2755136. doi: 10.1080/0886022X.2026.2755136 (PMC13757918; doi:10.1080/0886022X.2026.2755136)
Supplement: Supplementary file 1 [file IRNF_A_2755136_SM0046.docx]

Supplementary Table 1. Comparison of baseline characteristics between dropouts and completers in the experimental group

| Characteristics | Dropouts (n=6) | Completers (n=34) | *P* value |
| --- | --- | --- | --- |
| Age (year, $\overline{x}$± s) | 54.670 ± 13.049 | 54.088 ± 11.052 | 0.909 |
| Sex (n, male/female) | 4/2 | 23/11 | 1.000 |
| Body mass index (kg/m^2^, $\overline{x}$± s) | 25.065 ± 2.986 | 25.011 ± 3.029 | 0.968 |
| CKD Stage (n, Stage 1/2/3) | 3/0/3 | 10/10/14 | 0.340 |
| Systolic blood pressure (mmHg, $\overline{x}$± s) | 129.000 ± 8.099 | 126.190 ± 10.400 | 0.517 |
| Fasting plasma glucose (mmol/L, (M, IQR)) | 6.355, 2.070 | 6.480, 2.120 | 0.810 |
| 2-hour plasma glucose (mmol/L, (M, IQR)) | 10.000, 1.300 | 10.000, 2.250 | 0.644 |
| Glycated hemoglobin (%,(M, IQR)) | 7.450, 1.780 | 7.150, 2.030 | 0.671 |
| 24-hour urinary protein (g/d, (M, IQR)) | 4.125, 1.463 | 3.440, 3.080 | 1.000 |
| UACR (mg/g, (M, IQR)) | 2762.690, 2957.720 | 2587.230, 1613.420 | 0.868 |
| Albumin (g/L, (M, IQR)) | 32.150, 13.210 | 34.710, 7.700 | 0.383 |
| Blood urea nitrogen (mmol/L, (M, IQR)) | 8.015, 1.560 | 8.090, 5.215 | 0.671 |
| Serum creatinine (μmol/L, (M, IQR)) | 97.000, 75.500 | 108.000, 62.750 | 1.000 |
| Cystatin C (mg/L, (M, IQR)) | 2.003, 1.058 | 1.660, 0.883 | 0.517 |
| Serum uric acid (μmol/L, (M, IQR)) | 376.000, 72.000 | 387.000, 90.000 | 0.698 |
| eGFR (mL/min/1.73 m², (M, IQR)) | 61.310, 61.620 | 60.445, 52.000 | 0.644 |
| Triglycerides (mmol/L, (M, IQR)) | 1.980, 1.530 | 1.690, 1.905 | 0.985 |
| Total cholesterol (mmol/L, (M, IQR)) | 5.800, 0.980 | 5.030, 1.855 | 0.159 |
| Fibrinogen (g/L, (M, IQR)) | 4.480, 2.020 | 4.890, 1.970 | 0.644 |
| Erythrocyte sedimentation rate (mm/h, (M, IQR)) | 32.500, 18.750 | 34.000, 31.000 | 0.926 |
| C-reactive protein (mg/L, (M, IQR)) | 0.950, 2.760 | 1.280, 2.658 | 0.470 |
| Helper T-cell count (cells/μL, (M, IQR)) | 632.795, 258.690 | 623.940, 367.370 | 0.271 |
| Immunoglobulin G (g/L, (M, IQR)) | 8.230, 6.550 | 9.860, 3.970 | 0.698 |
| Metformin Administration (n, (Yes, No)) | 4, 2 | 19, 15 | 1.000 |
| SGLT2 Inhibitor Administration (n, (Yes, No)) | 5, 1 | 32, 2 | 0.394 |
| GLP-1R Agonist Administration (n, (Yes, No)) | 1, 5 | 4, 30 | 1.000 |
| DPP-4 Inhibitor Administration (n, (Yes, No)) | 0, 6 | 5, 29 | 1.000 |
| Insulin Administration (n, (Yes, No)) | 2, 4 | 18, 16 | 0.661 |
| Atorvastatin Administration (n, (Yes, No)) | 4, 2 | 20, 14 | 1.000 |
| MRA Administration (n, (Yes, No)) | 0, 6 | 0, 34 | - |

Annotations: CKD, chronic kidney disease; UACR, urinary albumin-to-creatinine ratio; eGFR, estimated glomerular filtration rate; SGLT2, sodium-glucose cotransporter 2; GLP-1R, glucagon-like peptide-1 receptor; DPP-4, dipeptidyl peptidase-4; MRA, mineralocorticoid receptor antagonist.

Supplementary Table 2. Components of the renal composite endpoint

| Component | Control group (n) | Experimental group (n) |
| --- | --- | --- |
| ESRD | 3 |  |
| Doubling of serum creatinine | 6 | 4 |
| Renal-related death | 1 |  |
| Other causes of death | 3 |  |
| Total | 13 | 4 |

Supplementary Table 3. Longitudinal changes in systolic blood pressure, HbA1c, and SGLT2 inhibitor use during follow-up

|  | Systolic blood pressure  (mmHg, $\overline{x}$± s) | | |  | Glycated hemoglobin  (%, (M, IQR)) | | |  | Sodium-glucose cotransporter inhibitor use (n, %) | | |
| --- | --- | --- | --- | --- | --- | --- | --- | --- | --- | --- | --- |
|  | Control group | Experimental group | *P* value |  | Control group | Experimental group | *P* value |  | Control group | Experimental group | *P* value |
| Baseline | 129.150 ± 14.913 | 126.600 ±9.668 | 0.367 |  | 7.450, 2.725 | 7.150, 1.750 | 0.754 |  | 36, 90.00 | 37, 92.50 | 1.000 |
| 3 month | 129.875 ± 8.237 | 127.800 ± 13.233 | 0.398 |  | 7.000, 0.850 | 7.000, 1.000 | 0.403 |  | 35, 94.59 | 18, 90.00 | 0.607 |
| 6 month | 130.275 ± 9.408 | 129.000 ± 8.987 | 0.537 |  | 6.700, 1.750 | 6.650, 0.975 | 0.554 |  | 33, 94.29 | 13, 86.67 | 0.574 |
| 9 month | 130.675 ± 7.727 | 128.550 ± 14.100 | 0.406 |  | 6.550, 1.000 | 6.600, 1.450 | 0.431 |  | 31, 93.94 | 9, 81.82 | 0.256 |
| 12 month | 130.175 ± 7.376 | 127.750 ± 9.475 | 0.205 |  | 6.300, 1.200 | 6.550, 1.000 | 0.126 |  | 27, 90.00 | 9, 81.82 | 0.598 |

Supplementary Figure 1. Effects of Kunxian capsule on inflammatory markers (A) erythrocyte sedimentation rate and (B) C-reactive protein levels
